# The Climate of Rome and the Roman Malaria

**Published:** 1893-06

**Authors:** 


*The Climate of Rome and the Roman Malaria.* By Professor TOMMASI-CRUDELI. Translated from the Italian by CHARLES CRAMOND DICK. Pp. 163. London: J. & A. CHURCHILL. 1892.

Professor Tommasi-Crudeli, who, after many years of hard work as a deputy of one of the colleges of his native province of Tuscany, has lately been made a Senator, delivered a set of lectures in 1885, when President of the Hygienic Institute in connection with the University of Rome, and those lectures are the basis of this work. He also delivered an address on the same subject at the International Medical Congress at Copenhagen in 1884.[1]

Every medical man who is likely to be consulted by patients going to Rome or any other part of Italy—and who in the present day is there that may not be so consulted?—should certainly read this work, as it combats many a preconceived idea as to the climate and water supply of Rome, about which there are undoubtedly erroneous notions as a rule. Professor Crudeli comes to the conclusion that Rome is healthier than many other cities of Europe, and would be the healthiest were it not for the malaria. The drinking-water supplies of Rome are shown to be good; four of the ancient sources are still being used and all are excellent in quality.

Professor Crudeli enters at great length into the composition and geological history of the soil of the different parts of the Agro Romano (Campagna), and the action of meteoric water upon it as it percolates through. His studies in this direction have clearly shown the great errors that have been made during the past twenty years in the many different methods for improvement taken in hand, especially as to cultivation. The great mistake he considers was made in ploughing the hills, which now have often become denuded of their thin covering of soil by its disturbance, and so an increase in the unhealthy swamps in the valleys is produced. Throughout the district it appears that there is an enormous volume of subterranean water lying in the numerous craters of extinct volcanoes and open basins formed by them; the geological formation is also to a great extent like a sponge soaked with water, the exit of which is prevented by many obstacles. So much is this the case, that on many hills to the right of the Tiber it is impossible to plant trees without providing a drain. It appears that the draining off of this water, a vast and difficult undertaking at which the ancients laboured, must be undertaken by the Italian Government. "It is necessary to isolate the ground of the Roman valleys, so that no other waters should penetrate therein, except the rain which falls directly upon it."

[1] *La Production Naturelle de la Malaria et les Assainissements des Terrains Malariques.* Par M. Tommasi-Crudeli.

A most interesting *resumé* of the history of malaria is given from the earliest period. It is shown also that the idea that existed that malaria was produced from stagnant water and marshy ground, through the putrefaction of dead animal and vegetable organic matter which they contain, is erroneous. Many a hill, far removed from water, marsh, or rice-bed, not only in Italy, but, as is well known by ourselves, also in India, is deadly with malaria. Malaria is produced in the earth, not in water. "The *sine quâ non* condition of the production of malaria is the existence of the malarious ferment in the earth." Then, the malarious soil must be kept damp during hot weather, or it will not produce malaria. Consequently, all conditions being equal, malarious regions where stagnant water is found are justly considered as more infected, because then the humidity indispensable to the production of malaria is never absent, even in the driest summer. It appears that the planting of eucalyptus trees is no good, and that the well-known trial made by the Trappist fathers at the convict station at Tre Fontane, a little way from Rome, has been a failure, the inhabitants having been severely attacked—so much so, that Professor Crudeli urged the Government to remove the convicts, which has since been done.

The use of arsenic is shown to be highly preventive; under its use slight attacks are cured by quinine. Quinine, however, may even aggravate an attack in chronic cases. A very simple and efficacious remedy, and a popular one, appears to be a decoction of a whole lemon.

Mr. C. C. Dick, who has resided a great deal in Italy and Rome for some years past, in occupying his leisure time by translating Professor Crudeli's book, has rendered a service to the medical profession in this country which might be well copied by others. Mr. Dick, possessing the friendship of Professor Crudeli, has had the advantage of his translation being revised by him. We have carefully compared it with the original Italian work, and find it most accurate. We can recommend everyone who is at all interested in the subject to read this extremely interesting and exhaustive work, which in its next edition we hope will be supplied with an index.

